# Porous Carbon Material Derived from Steam-Exploded Poplar for Supercapacitor: Insights into Synergistic Effect of KOH and Urea on the Structure and Electrochemical Properties

**DOI:** 10.3390/ma15082741

**Published:** 2022-04-08

**Authors:** Dayong Ding, Lan Ma, Xin Li, Zhong Liu, Lanfeng Hui, Fengshan Zhang, Yumeng Zhao

**Affiliations:** 1 School of Light Industry Science and Engineering, Tianjin University of Science and Technology, Tianjin 300457, China; 15631615922@sohu.com (L.M.); 934128141@mail.tust.edu.cn (X.L.); hlfeng@tust.edu.cn (L.H.); 2 Laboratory of Comprehensive Utilization of Paper Waste, Shandong Huatai Paper Co., Ltd., Dongying 257335, China; 3 National Engineering Laboratory for Pulp and Paper, China National Pulp and Paper Research Institute Co., Ltd., Beijing 100102, China; zhaoyumeng@cnppri.com

**Keywords:** porous carbon, KOH, urea, synergistic effect, supercapacitor

## Abstract

The electrochemical performance of supercapacitors using porous carbon as electrodes is strongly affected by the fabrication process of carbon material. KOH is commonly used as an activator combined with urea as a nitrogen dopant. However, the roles of KOH and urea in pore structure configuration and the electrochemical behavior of porous carbon electrodes are still ambiguous. Herein, the optimum porous carbon is obtained when KOH and urea are used simultaneously. KOH is used as a pore-forming substance, whereas urea is employed as a nitrogen source for the nitrogen doping of porous carbon, which increases its defect sites while reducing the graphitization degree. More importantly, urea also expands pores as a pore-enlarging agent, inducing interconnected porous structures. As a result, a hierarchical porous structure is formed and ascribed to the synergistic effect of KOH and urea, and the specific surface area reached 3282 m^2^ g^−1^ for sample PC800-4. The specific capacitance is 319 F g^−1^ at 0.5 A g^−1^ with excellent cycling stability over 2500 cycles. Furthermore, the symmetric supercapacitor reaches an excellent energy density of 11.6 W h kg^−1^ under 70.0 W kg^−1^ in a 6 M KOH electrolyte. Our work contributes to the rational designation of the porous carbon structure for supercapacitor applications.

## 1. Introduction

Supercapacitors have received substantial attention as current and promising energy storage systems used in burgeoning portable devices, smart power sources, and electric vehicles [1,2]. Porous carbon materials have attracted extensive interest as a potential electrode material for in high-performance supercapacitors with the advantages of great safety, high specific surface area, abundant pore structure, and excellent electrical and thermal conductivity [3,4]. The capacitive performance of porous carbon electrodes was limited by the structure and properties of precursor, heteroatom doping, and porous structures.

Compared with various precursors for preparing carbon materials, such as conductive polymers [5,6,7] and metal-organic frameworks [8], lignocellulosic biomass resources are attractive because of their low cost, abundance, and renewability. Lignocellulosic biomass is abundant, environmentally friendly and widely used as precursor for porous carbon materials [9,10]. However, lignocellulosic biomass exhibits an anti-degradation barrier and a compact structure that inhibits the activator’s penetration, rendering pretreatment of biomass precursors necessary. Pretreatment methods mainly include the hydrothermal method and heat treatment [11]; however, these methods are inefficient in dispersing fibers. Steam explosion pretreatment, as a combination of hydrothermal pretreatment and mechanical effects, has been widely used in lignocellulosic biomass pretreatment in the biorefinery industry [12]. Steam explosion pretreatment deconstructs biomass cell walls, which induces dispersed fibers, loosens internal cell wall structures, and acquires porous biomass, facilitating the penetration of the activator so that it can disperse uniformly in the precursor.

Besides the rational selection of precursors, introducing heteroatoms to carbon materials is critical for improving the capacitive performance of carbon electrode materials. N [13], P [14], and S [15] have usually been doped to improve the capacitive properties of carbon materials, mainly conductivity and wettability. N is one of the most used atoms in increasing the capacitive performance of carbon materials and introduces additional pseudocapacitance and quantum capacitance [16,17]. Currently, the mechanism to achieve both porous structures and defect engineering is seldom reported, though biomass porous carbon materials provide promising results under challenging circumstances.

In this study, we propose a two-step strategy using the solid residue of steam-exploded poplar (SEP) as a porous self-template precursor. KOH was used to activate the pre-carbonization step, and urea was added in the step of high-temperature carbonization as a nitrogen source to integrate both activation and heteroatom doping to produce biomass-derived porous carbon electrodes with high capacitive performance. The physicochemical and electrochemical properties of porous carbon prepared by the two-step activation were investigated. The mechanism of the synergetic effect of KOH and urea on the porous structure of the carbon material was also explored.

## 2. Materials and Methods

### 2.1. Preparation of Porous Carbon Materials

The steam-exploded poplar (the yield is about 65% calculated from oven dried-poplar) was used as a precursor and crushed in a portable high-speed grinder at a constant speed of 25,000 r/min. The required 40–60 mesh samples were screened out. For a typical routine, 1.0 g of SEP and 0–2.0 g KOH were added to 10 mL of deionized water and retained for 2 h under room temperature. Then, the mixture was dried at 105 °C in an oven and heated at 450 °C (pre-carbonization) in a tube furnace under flowing N_2_. The pre-carbonized carbon was obtained after grinding. 0–1.5 g urea and 1.0 g of pre-carbonized product were mixed and dissolved in 10 mL of deionized water. The mixture was reacted at room temperature for 2 h and dried at 105 °C in an oven. Afterward, the sample was carbonized at 800 °C in a tube furnace under flowing N_2_. Finally, black powder was obtained and washed with anhydrous ethanol, 2 M hydrochloric acid, and deionized H_2_O several times to neutralize. After drying, the porous carbon (PC) sample was obtained, and the yield was about 20% (calculated from steam-exploded poplar).

To reveal the synergistic effects of KOH and urea on porous carbon, porous carbon samples were prepared and later compared without KOH and urea (PC800-1), with only KOH (PC800-2), with only urea (PC800-3), and with both KOH and urea (PC800-4). The physicochemical and electrochemical properties of the porous carbon samples were analyzed.

### 2.2. Characterization

Scanning electron microscopy (SEM, LEO 1530VP) and transmission electron microscopy (TEM, Talos G2 200X) were performed to obtain the morphology. Raman spectra were obtained from a Raman spectrometer (Edinburgh RM5 XploRA). The specific surface area was investigated using the Brunauer–Emmette–Teller (BET) method; pore size distribution was determined by density functional theory (DFT) using Autosorb iQ. X-ray photoelectron spectroscopy (XPS) analysis was conducted with an X-ray photoelectron spectrometer (Thermo ESCALAB 250XI). The crystallinity of the prepared material was analyzed using X-ray diffraction (XRD) measurement (Ultima IV). TA Instruments’ Q50 analyzer was used to perform thermogravimetric analysis (TGA).

### 2.3. Electrochemical Measurements

The porous carbon electrode was placed into a three-electrode system in a 6 M KOH aqueous solution with a Pt electrode as the counter electrode, with the Hg/HgO electrode serving as the reference electrode. The working electrode was fabricated by mixing porous carbon (80%), a conductive agent (acetylene black, 10%), and binder (polyvinylidene fluoride, PVDF, 10%). The obtained slurry was then placed in a dispersant (N-methyl pyrrolidone, NMP). After mixing evenly, it was coated onto a 1 × 1 cm foam nickel sheet and dried in an oven at 80 °C to obtain the electrodes. The loading mass of the porous carbons was 3 mg.

Cyclic voltammetry (CV), galvanostatic charge/discharge (GCD) tests, and electrochemical impedance spectroscopy (EIS) were conducted in an electrochemical workstation of CHI660E (Shanghai Chenhua Instruments Co. Ltd., Shanghai, China). A CV test was performed within the range of −1–0 V.

The specific gravimetric capacitance of a single electrode (F g^−1^) determined from the galvanostatic cycles was calculated by means of the formula:

(1) $$C_{g}=\frac{I\cdot \Delta t\;}{m\cdot \Delta V}$$

where *I* is the applied current, *Δt* is the discharge time, *ΔV* is the voltage change, and *m* is the mass of porous carbon, respectively.

The electrochemical performance of the PC800-4 was further investigated using a two-electrode system. A symmetric supercapacitor device was assembled with two PC800-4 electrodes separated by a cellulose membrane (NKK, TF4030) in the aqueous KOH solution electrolyte (6 M KOH). The energy density (*E*, Wh kg^−1^) and power density (*P*, W kg^−1^) were determined based on GCD curves from the following equations [18]:

(2) $$E=\frac{0.5CV^{2}}{3.6}$$

(3) $$P=\frac{3600E}{t}$$

where *t* is the discharge time (s).

## 3. Results

### 3.1. Effect of KOH Dosage on the Porous Structure and Electrochemical Performance of Carbons in the Pre-Carbonization Process

**Figure 1:**
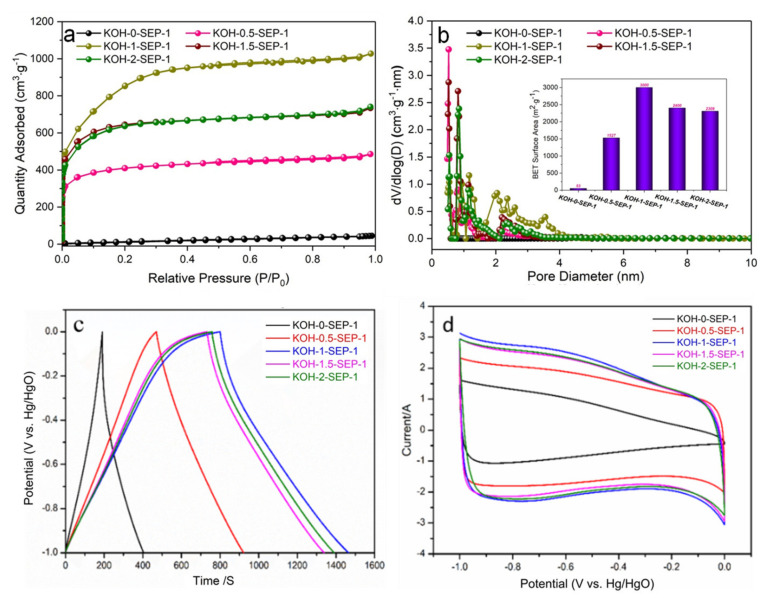
Nitrogen adsorption–desorption isotherms of pre-carbonized carbons prepared under different KOH/SEP ratios at 450 °C (**a**). Pore size distribution (**b**). GCD curves of various KOH ratios at a current density of 0.5 A g^−1^ (**c**). CV curves of various KOH ratios at a scan rate of 10 mV s^−1^ (**d**).

BET isotherms of pre-carbonized carbons prepared under different KOH/SEP ratios at 450 °C are shown in Figure 1a. The carbons presented adsorption isotherm of type I [19], and the nitrogen adsorption capacity of all samples increased rapidly when the relative pressure (P/P^0^) was low, indicating that the material was rich in micropores. When the relative pressure increased sequentially, the growth rate of adsorption slowed down. Meanwhile, the adsorption capacity of the sample increased with the increase in KOH/SEP ratio. When the ratio of KOH/SEP was 1, the carbon reached maximum adsorption capacity, indicating that increasing the amount of KOH strengthened the activation effect and further developed the porous structure, thereby increasing the micropores in the sample, as shown in Table 1. However, when the KOH/SEP ratio increased to 2, the adsorption capacity of the carbon decreased slightly at relatively low pressures, suggesting that excessive KOH dosage may lead to excessive activation, destroying the pore structure and reducing the micropores.

Pore size distribution was obtained through the DFT method (Figure 1b). Combined with the pore structure parameters shown in Table 1, the pore size of carbon with a KOH/SEP ratio of 0.5 was mainly in the range of 0.5–2.0 nm. When increasing the KOH/SEP ratio, the pore size distribution of samples with KOH/SEP ratios of 1, 1.5, and 2 became wider. Our results showed that the increase in KOH addition played a pivotal role in pore expansion. The pore structure parameters of pre-carbonized carbons in Table 1 showed that when the KOH/SEP ratio was 1, it exhibited the largest specific surface area and total pore volume, which were 3000 m^2^/g and 1.59 cm^3^/g, respectively. The ultrahigh, specific surface area was beneficial to accumulating more electrolyte ions and improving the capacitance of poplar-based porous activated carbon.

The electrochemical performance of pre-carbonized carbons in the KOH three-electrode system was tested. Figure 1c shows the GCD curves of carbons at 0.5 A g^−1^. The GCD curve formed a slightly twisted symmetrical triangle, declaring that the capacitance was primarily an electric double-layer capacitance. The discharge curve was slightly distorted, indicating the presence of some pseudo-capacitance. The CV curves in Figure 1d are almost rectangular, indicating that the sample had electrical double-layer capacitance characteristics. A wide oxidation-reduction peak was observed between −1 and 0.5 V, demonstrating that the carbons also contained pseudo-capacitance in accordance with the CV results. These pseudo-capacitances were mainly derived from the fast redox reaction of rich heteroatoms in P450s [20]. Among the five samples, pre-carbonized carbons prepared with a KOH/SEP ratio of 1 had the largest CV curve area, and the calculated specific capacitance was 360 F g^−1^. According to Table 1, the highest capacitance value of the porous carbon can be ascribed to its high specific surface area (3000 m^2^/g), which introduces more active sites for the accumulation of electrolyte ions. Consequently, the optimal ratio of KOH/SEP is 1.

### 3.2. Effect of Urea Dosage on the Porous Structure and Electrochemical Performance of Carbons in the Carbonization Process

**Figure 2:**
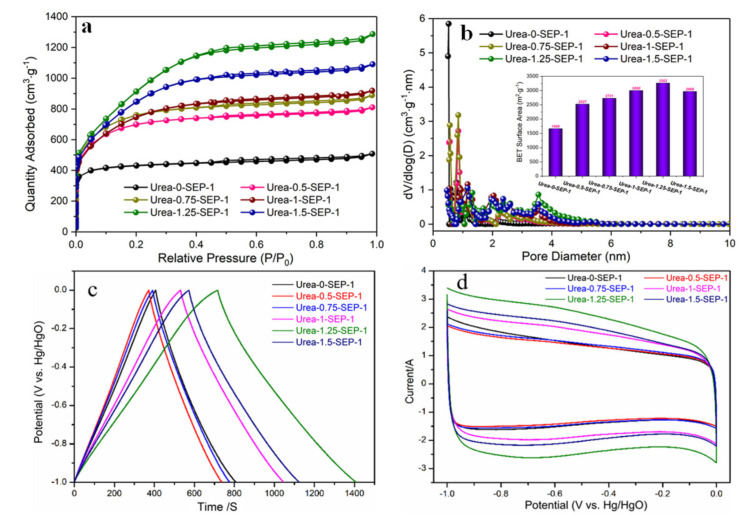
Nitrogen adsorption–desorption isotherms of porous carbons prepared with various urea/SEP ratios at 800 °C (**a**). Pore size distribution (**b**). GCD curves at various urea ratios at a current density of 0.5 A g^−1^ (**c**). CV curves at various urea ratios at a scan rate of 10 mV s^−1^ (**d**).

BET isotherms of porous carbons prepared under various urea/SEP ratios at 800 °C are shown in Figure 2a. The carbons presented a type I adsorption isotherm, and the nitrogen adsorption capacity of all samples increased rapidly when the relative pressure (P/P0) was low, indicating that the material was rich in micropores. When the relative pressure increased, the growth rate of adsorption slowed down. The horizontal platform indicated that the material’s pore structure was mainly micropores. The adsorption capacity of the samples increased gradually with the increase of the urea/SEP ratio at relatively low pressures. When the urea/SEP ratio was 1.25, the carbon exhibited its maximum adsorption capacity, indicating that the pore structure further increased with increased urea addition. However, when the ratio of urea/SEP increased to 1.5, the adsorption capacity of porous carbon with a urea/SEP ratio of 1.5 decreased at relatively low pressures, indicating that the excess urea addition may lead to excessive activation destroying the porous structure and reducing micropores.

We obtained the pore size distribution curves using the DFT method (Figure 2b). Together with Table 2, the pore size of porous carbon with a urea/SEP ratio of 0.75 was in the range of 0.5–2.0 nm. With the increase in the ratio of urea/SEP, the pore size distributions of porous carbon with urea/SEP ratios of 1, 1.25, and 1.5 became wider, involving 1–2 nm and 2–4 nm pores. According to the pore structure properties of porous carbons in Table 2, the specific surface area and total pore volumes of porous carbon prepared with a urea/SEP ratio of 1.25 were 3262 m^2^/g and 1.99 cm^3^/g, respectively.

The electrochemical performance of porous carbons in the KOH three-electrode system was also conducted. Figure 2c shows the GCD curves of carbons at 0.5A g^−1^. The curves showed a symmetrical triangle, indicating that the capacitance was primarily an electric double-layer capacitance, but the discharge curve was slightly distorted, indicating the effect of pseudocapacitance. Figure 2d showed the CV curves of porous carbons at the scanning rate of 10 mV/s, and the potential window was −1.0–0 V. The CV curves of porous carbons were almost rectangular, indicating that the samples had electric double-layer capacitance characteristics. A wide oxidation-reduction peak emerged between −1 and 0.5 V, showing that porous carbons also contained pseudocapacitance consistent with the CV results. Among the six samples, the CV curve area of porous carbon prepared with a urea/SEP ratio of 1.25 was the largest and had the highest specific capacitance (351 F g^−1^). According to Table 2, the highest capacitance of the carbon can be ascribed to the large specific surface area (3262 m^2^/g), which provided many active sites for the accumulation of electrolyte ions. In conclusion, the optimal urea/SEP ratio was 1.25.

### 3.3. Morphology and Structure of PCs

**Figure 3:**
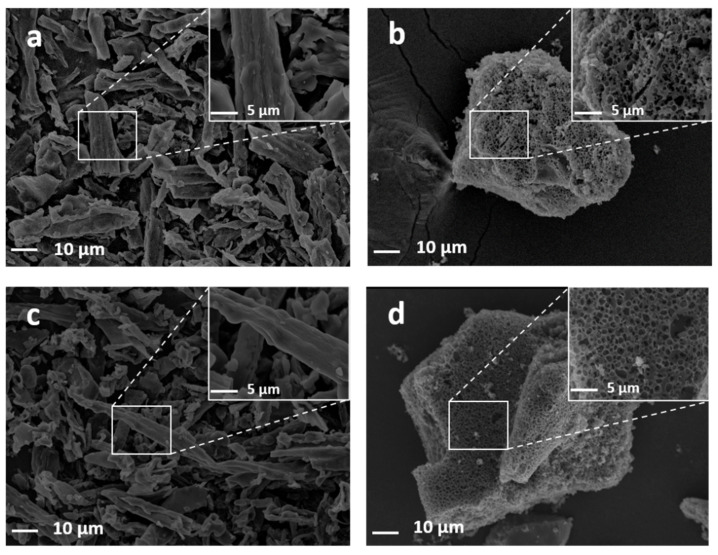
SEM images of PC800-1 (**a**), PC800-2 (**b**), PC800-3 (**c**), and PC800-4 (**d**).

The SEM images of porous carbon samples are shown in Figure 3. The irregular honeycomb structure of porous carbon can be observed in Figure 3b,d when KOH was used as an activator. When only urea was used, the PC800-3 was fibrous without a porous structure (Figure 3c). Therefore, these pore structures may be attributed to the activation of KOH. The pores formed by KOH and urea together (Figure 3d) demonstrated more cellular structures of porous carbon than those formed using only KOH. However, when urea was used without KOH, the effect on the fiber was not prominent. Therefore, the coexistence of the activator (KOH) and urea worked synergistically.

**Figure 4:**
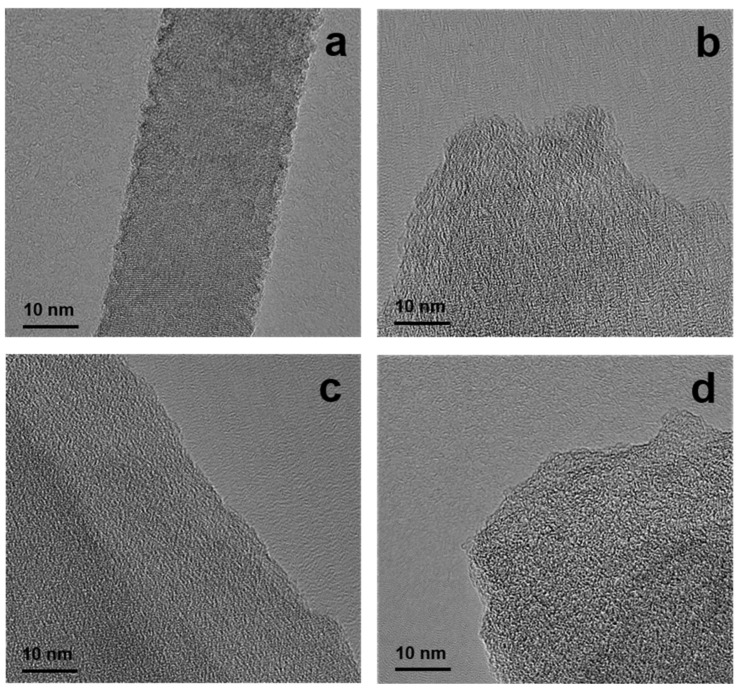
TEM images of PC800-1 (**a**), PC800-2 (**b**), PC800-3 (**c**), and PC800-4 (**d**).

The nanostructure images were obtained by TEM. When the magnification was 400,000, the pore structure in the porous carbon could be observed, and these pore knots formed a worm-like shape. In addition, the pore size of PC800-4 (Figure 4d) was significantly larger than that of PC800-1 (Figure 4a), which may be due to the reaction between ammonia gas produced by urea pyrolysis, carbon, and the etching of carbon [21,22]. As a result, the amorphous region of porous carbon materials increased.

**Figure 5:**
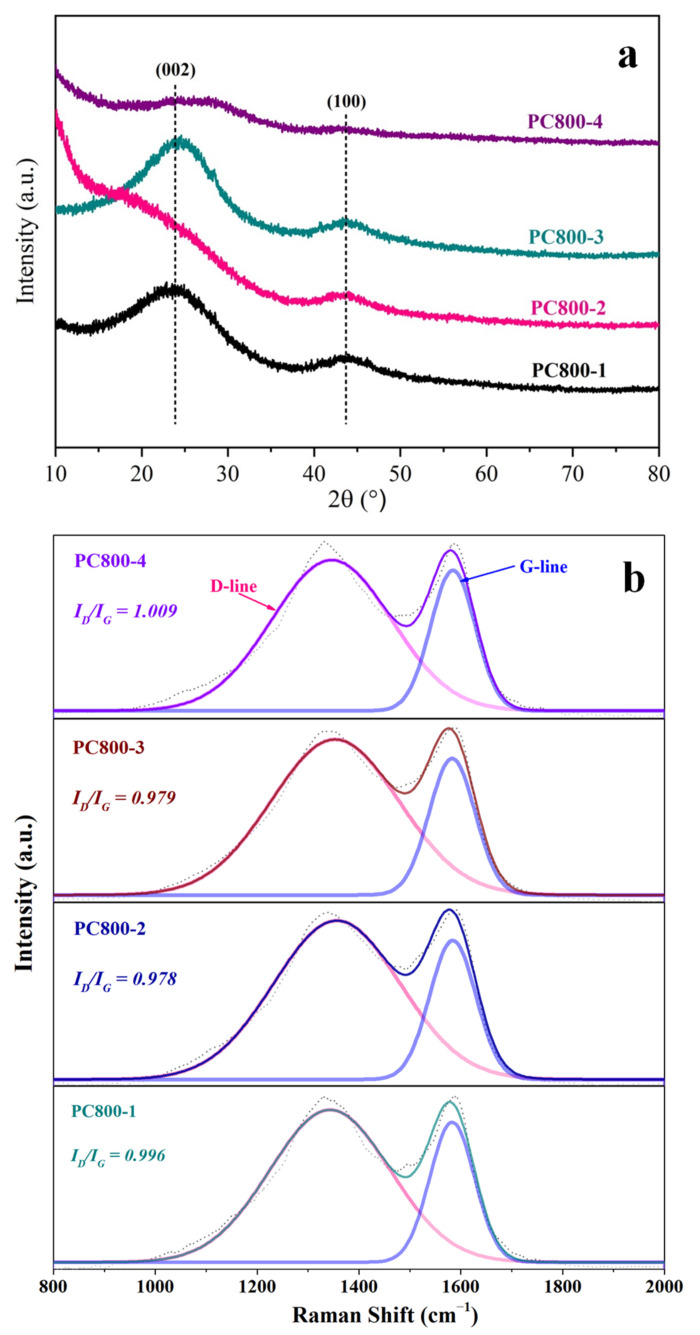
XRD patterns (**a**) and Raman spectra (**b**).

The graphitization degree of PC800s was analyzed by X-ray diffraction and Raman spectroscopy. Figure 5a shows the XRD pattern of PC800s. Two wide characteristic peaks at 23° and 43.8° correspond to (002) and (100) planes of amorphous carbon [14]. Compared with PC800-2 and PC800-4, the two characteristic peaks of PC800-1 and PC800-3 were sharper, indicating that PC800-1 and PC800-3 possessed higher graphitization degrees. However, with the addition of KOH, the diffraction peaks of PC800-2 and PC800-4 flattened, suggesting the weakening of their graphitization degrees. The amount of KOH also had a stronger etching effect on the framework of carbon samples, breaking their ordered graphite flakes and forming more porous structures. To further determine the graphitization structure of SEP-graded porous carbon under different conditions, the Raman spectra of PC800s were recorded; the results are shown in Figure 5b. The two characteristic peaks of porous carbon were located at 1320 cm^−1^ (D peak) and 1580 cm^−1^ (G peak). The D peak at 1320 cm^−1^ represents the disorder degree of sp^2^-hybridized carbon, where the catalyst can form active defect sites. The G peak at 1580 cm^−1^ represents the graphitization degree of porous carbon. The strength of the D peak indicates the microcrystalline size in graphite [23]. The intensity ratio of the D–G peak (I_D_/I_G_) is used to characterize the defect degree of heteroatom-doped carbon-based materials [24]. The ratio for each sample was close to 1, indicating that the SEP-based porous carbon prepared under different conditions was amorphous with a certain degree of graphitization. When urea was not added, the I_D_/I_G_ of porous carbon (PC800-2) was 0.978. After adding urea, the I_D_/I_G_ of porous carbon (PC800-4) slightly increased to 1.009 because the formation of nitrogen-doped carbon increased the number of defects [25]. When KOH and urea were used simultaneously, the number of defects increased, and the graphitization degree decreased, improving the electrochemical performance of porous carbon.

**Figure 6:**
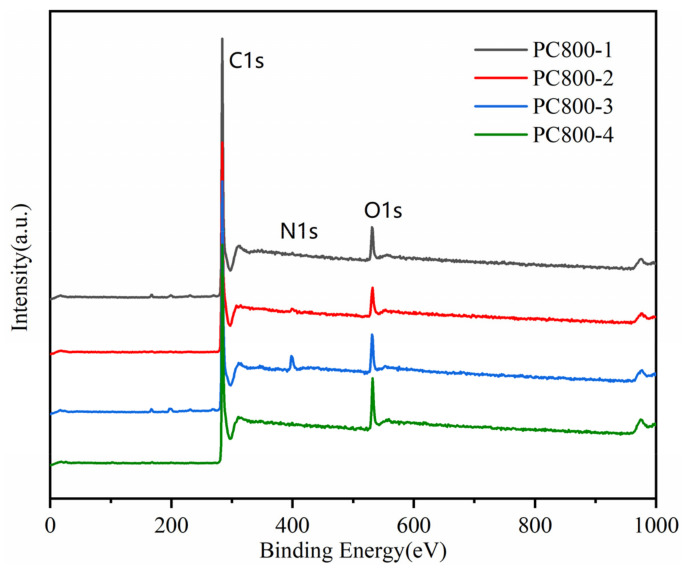
XPS spectra of PC800-1, PC800-2, PC800-3, and PC800-4.

**Figure 7:**
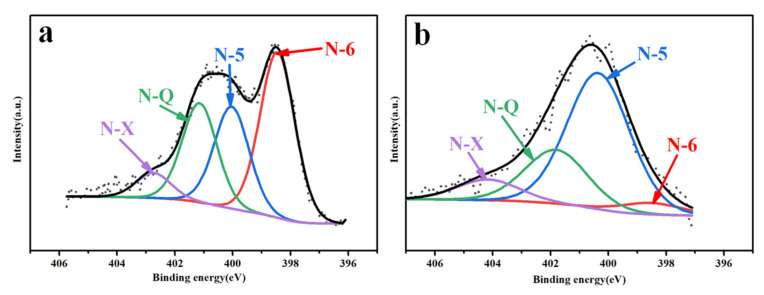
High-resolution N1 s spectra of PC800-3 (**a**), PC800-4 (**b**).

The surface chemical characteristics of PC800s were analyzed by XPS (Figure 6). Two sharp peaks representing C1s and O1s peaks were detected. The surface elements of the samples were C, O, and N (284.7 eV, 533 eV, and 401 eV, respectively) [26,27]. The specific contents of the three elements are shown in Table 3. The N1s peak strengths of PC800-1 and PC800-2 were weak, indicating that the nitrogen content was low due to the absence of urea. The nitrogen contents of PC800-3 and PC800-4 were 5.84 and 1.76 (atom %), respectively. When KOH activated the sample, the nitrogen content decreased significantly. This may be due to the reaction of biuret, melamine, cyanuric amide, or isocyanate with KOH during the pyrolysis of urea. The C1s spectra of PC800s is shown in Figure S1, including the sp2-C (~284.7 eV) and oxygen-containing functional groups C-O (~286.6 eV) and O=C-O (~288.5 eV). Figure 7 shows the N1s spectra of PC800-3 and 4 (the N contents of PC800-1 and 2 were low due to the lack of urea addition, inducing a poor signal-to-noise ratio. Data not shown), including pyridine nitrogen (N-6, ~398.7 eV), pyridine/pyrrole nitrogen (N-5, ~400.3 eV), graphite nitrogen (N-Q, ~401.4 eV), and nitrogen oxide (N-X, ~403 eV) [28]. It corresponds to the N atoms doped at the edge (pyridine-N) and central (graphite-N) regions of the graphite layer. As presented in Table 4, most of the nitrogen atoms were graphite nitrogen, and the relative content of graphite nitrogen increased from 0.49 to 0.68, and the pyridine nitrogen decreased from 1.59 to 0.04 with the addition of KOH. KOH activation destroyed the graphite layer and exposed more graphite edges in the carbon skeleton, increasing graphite nitrogen and decreasing pyridine nitrogen [29]. Graphite nitrogen improves the electronic conductivity of the carbon skeleton, and pyridine nitrogen can introduce the pseudocapacitance effect [30,31]. Therefore, compared with PC800-3, PC800-4 exhibited better electrochemical performance and further demonstrated KOH and urea’s synergistic effect.

We found visible differences in the pyrolysis curves by comparing the pyrolysis curves of different mixtures and pre-carbonization products (Figure S2)**.** The weight loss of the urea mixture and pre-carbonization products was the highest due to the thermal decomposition of urea. However, the pyrolysis curve of the mixture was significantly different from that of P450 + urea when the same proportion of urea was added (P450 + KOH + urea). From room temperature up to 373 °C, an obvious weight loss peak was obtained at relatively lower temperatures (room temperature up to 215 °C). Therefore, we speculated that one or more products of biuret, melamine, cyanuric amide, or isocyanate in the pyrolysis products acted on carbon together with KOH to produce synergistic activation, increasing the specific surface area of porous carbon.

N_2_ adsorption-desorption was conducted to measure the porosity of the samples (Figure S3). Figure S3a shows the BET isotherms of PC800s prepared under different conditions. All isotherms of PC samples presented type I. The adsorption capacity of all the samples increased rapidly under low relative pressure (P/P^0^), indicating that the materials had abundant micropores. With the increase in relative pressure, the adsorption growth rate slowed down, and an approximate level platform appeared, indicating that the type of material pores was mainly micropores. The adsorption capacity of the sample increased with the addition of KOH and urea at relatively low pressures. When KOH and urea were added simultaneously, PC800-4 had the maximum adsorption capacity, indicating that KOH and urea acted synergically, increasing the micropores in the sample. The relevant porous parameters are provided in Table 5. PC800-4 exhibited the highest specific surface area of 3282 m^2^·g^−1^ due to the synergetic effect of KOH and urea. Figure S3b shows the pore size distribution. Combined with the pore structure parameters shown in Table 5, the pore size distribution of PC800-4 was between 0.5 and 2.0 nm. After the addition of KOH and urea, the pore size distribution of PC800-1, PC800-2, and PC800-3 widened, and 1–2 nm and 2–4 nm pores increased. Our results showed that the addition of KOH and urea led to the expansion of the pores, indicating the formation of connected pores. The introduction of the interconnected mesopores forms ion channels and improves sample rate performance [32].

### 3.4. Electrochemical Performance of Carbon Electrodes

**Figure 8:**
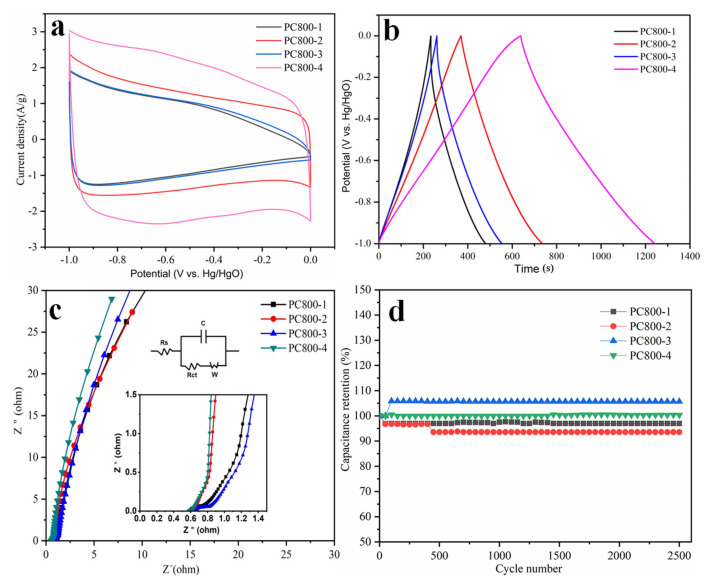
Electrochemical performance of PC800s: cyclic voltammetry (CV) curves at 10 mV/s (**a**); galvanostatic charge/discharge (GCD) curves at a current density of 0.5 A g^−1^ (**b**); Nyquist plots of PC800s from 10 kHz to 10 mHz (**c**); capacitance retention (%) at a current density of 20 A g^−1^ (**d**).

The electrochemical test results of PC800s are shown in Figure 8. Figure 8a exhibits the CV curves of porous carbon materials prepared under four different conditions at a scanning rate of 10 mv s^−1^. The results showed that the CV curves of PC800-2 and PC800-4 are approximately rectangular, confirming that the porous carbon electrode demonstrated electric double-layer capacitance in the presence of KOH. When the voltage was in the range of −1–0.5 V, an oxidation-reduction peak indicating pseudocapacitance was also obtained. Due to the abundance of heteroatoms in carbon materials, the existence of heteroatoms can undergo redox reactions, increasing their pseudocapacitance properties [18]. PC800-4 had a larger CV area than PC800-2, indicating that its specific capacitance was higher than PC800-2. The CV areas of PC800-1 and PC800-3 were smaller than those of PC800-2 and PC800-4, and no redox reaction peak was obtained, suggesting their low pseudocapacitance performance. This result may be due to a reduction in the effective oxygen-containing functional groups PC800-1 and PC800-3. The reduced capacitances of PC800-1 and PC800-3 were ascribed to the absence of KOH.

The GCD curves of the samples prepared under four different conditions at 0.5 A g^−1^ are shown in Figure 8b. The charge-discharge curves of PC800-2 and PC800-4 are approximately symmetrical, indicating that the capacitance of poplar-based porous carbon material prepared with KOH was mainly electric double-layer capacitance [33]. However, the discharge curve of the prepared porous carbon material was slightly bent, indicating pseudocapacitance in the porous carbon material [34]. The discharge curve of PC800-4 was more bent than that of PC800-2, indicating that the addition of urea introduced the heteroatoms to the porous carbon material, increasing its pseudocapacitance and capacitance. The discharge curve of PC800-4 exhibited the longest discharge time, indicating that PC800-4 had the largest specific capacitance (319 F g^−1^ at 0.5 A g^−1^). The discharge curves of PC800-1 and PC800-3 were almost the same, indicating that the effect of urea on the specific capacitance was limited. When considering the discharge curves of PC800-3 and PC800-4, the specific capacitance of the samples could have significantly improved if the KOH and urea were both added. The specific capacitances of PC800s under different current densities were also calculated and plotted in Figure S4. PC800-4 has the highest initial capacitance retention of 62.6%, with the current density increased from 0.1 to 20 A g^−1^, demonstrating good rate capability. The values were 46.5, 52.8, and 52.4% for PC800-1, PC800-2, and PC800-3, respectively. The excellent rate of performance was caused by the optimal hierarchically porous structure and N doping induced by the synergistic effect of KOH activation and urea addition.

An AC impedance test was conducted; the Nyquist plots of the carbons are displayed in Figure 8c. The curves of PC800-2 and PC800-4 are approximately vertical in the low-frequency area, indicating that they had little ion diffusion resistance and good capacitance performance [35]. The combined series resistance (Rs), which comprises the internal resistance of the electrolyte, electrode material, and the contact resistance between the electrode material and current collector, can be obtained from the EIS diagram expressed by the intercept of the real impedance (Z’) axis in the high-frequency area. The Rs of the PC800-1, PC800-2, PC800-3, and PC800-4 samples are 0.59, 0.60, 0.65, and 0.58, respectively. Thus, all samples exhibited small resistance values, indicating good conductivity, especially for PC800-4.

Figure 8D shows the long cycle test curves of porous carbon samples under different preparation conditions for 2500 current charge-discharge cycles at a current density of 20 A g^−1^. The capacitance retention of samples PC800-1 and PC800-2 (without urea addition) were 97 and 94% after 2500 cycles, respectively. The PC800-3 and PC800-4 electrodes presented excellent stability despite the slight increase in specific capacitance from 70 F to 74 F g^−1^ and 148 F to 152 F g^−1^ after 2500 cycles, respectively. The capacitance retention was 103% for sample PC800-4. The increased specific capacitance should be ascribed to the activation of the porous carbon electrode; in other words, the intercalation and de-intercalation of the ions in the electrodes were processed thoroughly after the original hundreds of cycles, producing efficient active sites in the electrodes and consequently promoting the capacitance [36]. The excellent stability of PC800-3 and PC800-4 can be ascribed to the urea addition’s blockage of active oxidation sites. Table 6 shows the specific capacitance and capacitance retention of carbon materials derived from various precursors.

**Figure 9:**
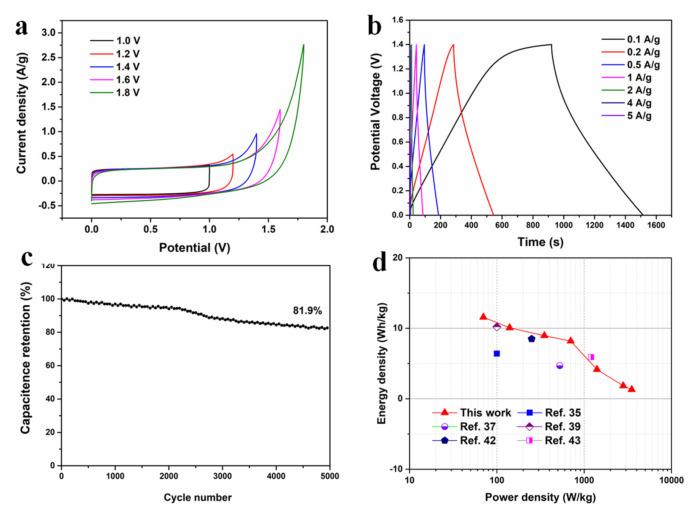
Electrochemical performance of PC800-4 electrode in a symmetric two-electrode system. (**a**) CV curves at the scan rates of 10 mV s^−1^. (**b**) GCD curves at the specific current densities of 0.1–5 A g^−1^. (**c**) Cyclic stability at a specific current of 5 A g^−1^ over 8000 cycles. (**d**) Ragone plot.

A symmetrical two-electrode system was fabricated to measure the electrochemical properties of PC800-4 in practical application. Figure 9a displays the CV curves for symmetric supercapacitor tests under 10 mV s^−1^ at different voltages in 6 M KOH. Rectangular-like shapes are still observed at the voltages of 0−1.6 V, indicating that a wide potential window of 0−1.6 V can be achieved. The GCD profiles at the potential window of 0−1.6 V display linearly symmetric triangular shapes (Figure 9b). The specific capacitance was 42.5 F g^−1^ at current densities of 1.0 A g^−1^. The cycling stability for the symmetric supercapacitor investigated reaches 81.9% at 5 A g^−1^ after 8000 cycles (Figure 9c), implying good electrochemical cycling stability. Furthermore, the PC800-4 symmetric supercapacitor displays an ultrahigh energy density of 11.6 W h kg^−^^1^ with 70 W kg^−^^1^ (Figure 9d) because of the multi-level pore structure and synergistic effect of an ultra-large specific surface area, which increases the contact area between the electrode and electrolyte. The result is comparable to or larger than some other porous carbons such as N-doped porous carbon nanofibers (energy density of 8.5 Wh kg^−1^ at a power density of 250 W kg^−1^) [42] and N-doped carbon nanofiber network (energy density of 5.9 Wh kg^−1^ at a power density of 1200 W kg^−1^) [43].

## 4. Conclusions

In summary, we presented a two-step carbonization method to produce N-doped porous carbon derived from steam-exploded poplar as a self-template precursor. KOH was used as an activator, and urea was added during the high-temperature carbonization process, serving as a nitrogen source and pore-enlarging agent. Profiting from the synergistic effect of KOH and urea, the prepared carbon material exhibited ultrahigh specific surface area, hierarchical porous network, high graphitization degree, and numerous nitrogen functional groups. The excellent structure characteristics described above endow the optimized PC800-4 electrode with an excellent specific capacitance, good conductivity, and superior capacitance stability in 6 M KOH electrolytes. The fabricated symmetrical two-electrode system device also showed good energy density and cycle stability. Our work can pave the way for practical applications of porous carbon in energy storage systems for electric double-layer capacitors, lithium-ion batteries, and fuel cells.

## Figures and Tables

**Table 1:** Pore characteristics of pre-carbonized carbons prepared under different KOH/SEP ratios at 450 °C and the specific capacitances of the corresponding samples.

Samples	S_BET_ (m^2^/g)	S_micro_ (m^2^/g)	S_meso_ (m^2^/g)	V_T_ (cm^3^/g)	Average Pore Size (nm)	Capacitance (F g^−^^1^)
KOH-0-SEP-1	53	-	53	0.07	4.82	95
KOH-0.5-SEP-1	1527	1233	294	0.75	1.97	230
KOH-1-SEP-1	3000	2012	988	1.59	2.13	360
KOH-1.5-SEP-1	2400	2081	319	1.14	1.89	333
KOH-2-SEP-1	2309	2010	299	1.15	1.99	346

**Table 2:** Pore characteristics of porous carbons prepared under various urea–SEP ratios at 800 °C and the specific capacitances of the corresponding samples.

Samples	S_BET_ (m^2^/g)	S_micro_ (m^2^/g)	S_meso_ (m^2^/g)	V_T_ (cm^3^/g)	Average Pore Size (nm)	Capacitance (F g^−1^)
Urea-0-SEP-1	1666	1511	155	0.79	1.88	201
Urea-0.5-SEP-1	2527	1998	528	1.25	1.98	180
Urea-0.75-SEP-1	2731	2004	726	1.38	2.02	193
Urea-1-SEP-1	3000	2012	988	1.59	2.13	262
Urea-1.25-SEP-1	3262	1307	1955	1.99	2.44	351
Urea-1.5-SEP-1	2968	1397	1572	1.69	2.28	278

**Table 3:** The C, O, and N contents of PC800s from XPS analysis.

Samples	C (Atom %)	N (Atom %)	O (Atom %)
PC800-1	93.00	0.57	6.43
PC800-2	89.99	0.49	9.52
PC800-3	87.07	5.84	7.09
PC800-4	91.91	1.76	6.34

**Table 4:** Contents of various types of N in PC800-3 and 4.

Samples	Pyridine-N (%)	Pyrrolidone-N (%)	Graphite-N (%)	Nitrogen Oxide (%)
PC800-3	1.59	3.42	0.49	0.34
PC800-4	0.04	0.93	0.68	0.11

**Table 5:** Porosity parameters of PC800s.

Samples	S_BET_ (m^2^/g)	S_micro_ (m^2^/g)	S_meso_ (m^2^/g)	V_micro_ (cm^3^/g)	V_meso_ (cm^3^/g)	V_T_ (cm^3^/g)
PC800-1	200	145	55	0.06	0.05	0.11
PC800-2	1412	1226	186	0.48	6.33	6.80
PC800-3	53	-	53	-	6.99	6.99
PC800-4	3282	1232	2050	1.16	0.95	2.11

**Table 6:** Comparison between specific capacitance and capacitance retention of carbon materials derived from various precursors.

Precursor	Doping Element	Capacitance (F g^−1^)	Electrolyte	Capacitance Retention (%)	Ref.
Polyacrylonitrile	N, P	224.9	1 M H_2_SO_4_	70	[35]
Glucose	N, P	183.8	6 M KOH	93.6	[37]
Formaldehyde	N, P	200	6 M KOH	91	[38]
Poly acrylic acid	N, S	309.4	1 M H_2_SO_4_	98.5	[39]
Melamine sponge	N	238	6 M KOH	99	[40]
Polyacrylonitrile	N	203.4	6 M KOH	>94	[41]
Poplar	N	319	6 M KOH	103	This work

## Data Availability

Not applicable.

## Supplementary Materials

The following supporting information can be downloaded at: https://www.mdpi.com/article/10.3390/ma15082741/s1, Figure S1: High-resolution C1s spectra of PC800-1 (a), PC800-2 (b), PC800-3 (c), and PC800-4 (d); Figure S2: TG/DTG curves of pre-carbonized SEP under different conditions in a flow of nitrogen gas; Figure S3: Nitrogen adsorption-desorption isotherms (a), Pore size distribution curves (b); Figure S4: Specific capacitances of the PC800s at different current densities (0.1–20 A g^−1^).
