# Supplementary material for: Porous Carbon Material Derived from Steam-Exploded Poplar for Supercapacitor: Insights into Synergistic Effect of KOH and Urea on the Structure and Electrochemical Properties
Source: Materials (Basel). 2022 Apr 8;15(8):2741. doi: 10.3390/ma15082741 (PMC9027541; doi:10.3390/ma15082741)
Supplement: Supplementary file 1 [file materials-15-02741-s001.zip › materials-1614380-supplementary.pdf]

*Supplementary Material*

# Porous Carbon Material Derived from Steam-Exploded Poplar for Supercapacitor: Insights into Synergistic Effect of KOH and Urea on the Structure and Electrochemical Properties

**Dayong Ding [1,2,*], Lan Ma [1], Xin Li [1], Zhong Liu [1,*], Lanfeng Hui [1], Fengshan Zhang [2,*] and Yumeng Zhao [3]**

[1] School of light industry science and engineering, Tianjin University of Science and Technology, Tianjin 300457, China; 15631615922@sohu.com (L.M.); 934128141@mail.tust.edu.cn (X.L.); hlfeng@tust.edu.cn (L.H.)

[2] Laboratory of Comprehensive Utilization of Paper Waste, Shandong Huatai Paper Co Ltd., Dongying 257335, China

[3] National Engineering Laboratory for Pulp and Paper, China National Pulp and Paper Research Institute Co. Ltd., Beijing 100102, China; zhaoyumeng@cnppri.com

* Correspondence: ddyong2019@tust.edu.cn (D.D.); mglz@tust.edu.cn (Z.L.); jszx@huatai.com (F.Z.)

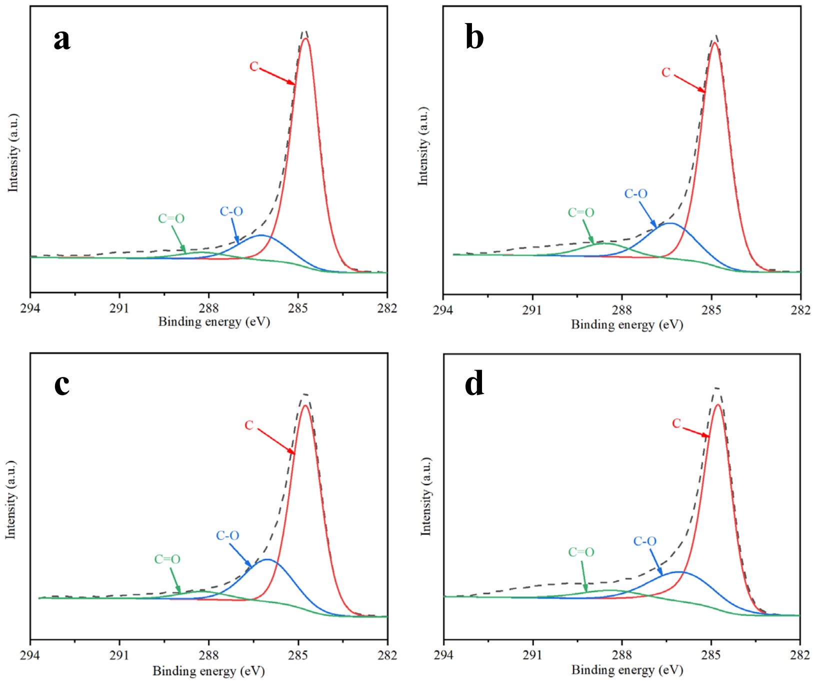


**Figure S1.** High-resolution C1 s spectra of PC800-1 (**a**), PC800-2 (**b**), PC800-3 (**c**), and PC800-4 (**d**).

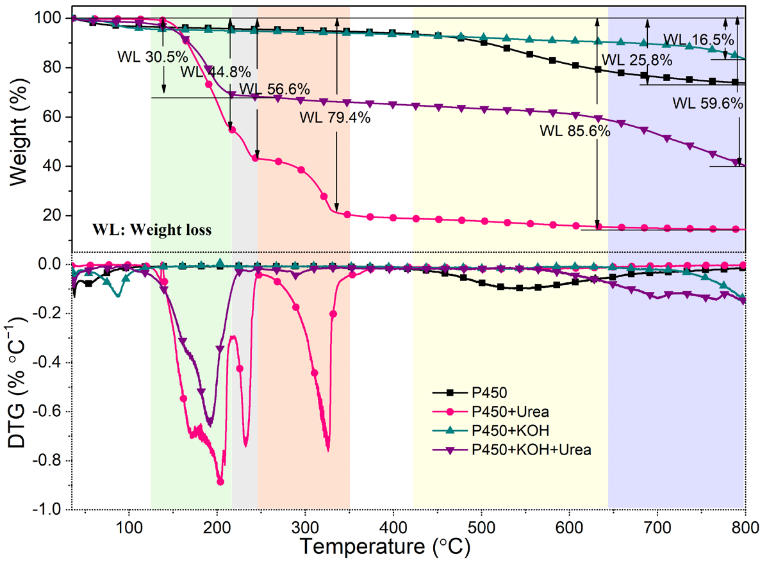


**Figure S2.** TG/DTG curves of pre-carbonization SEP under different conditions in a flow of nitrogen gas.

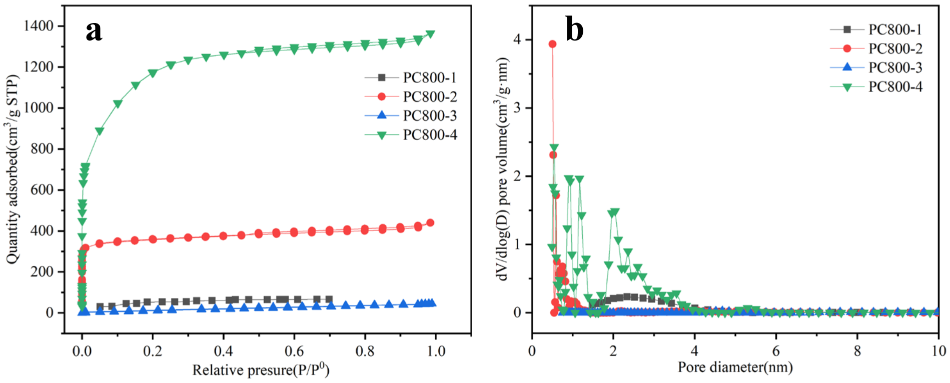


**Figure S3.** Nitrogen adsorption-desorption isotherms (**a**), Pore size distribution curves (**b**).

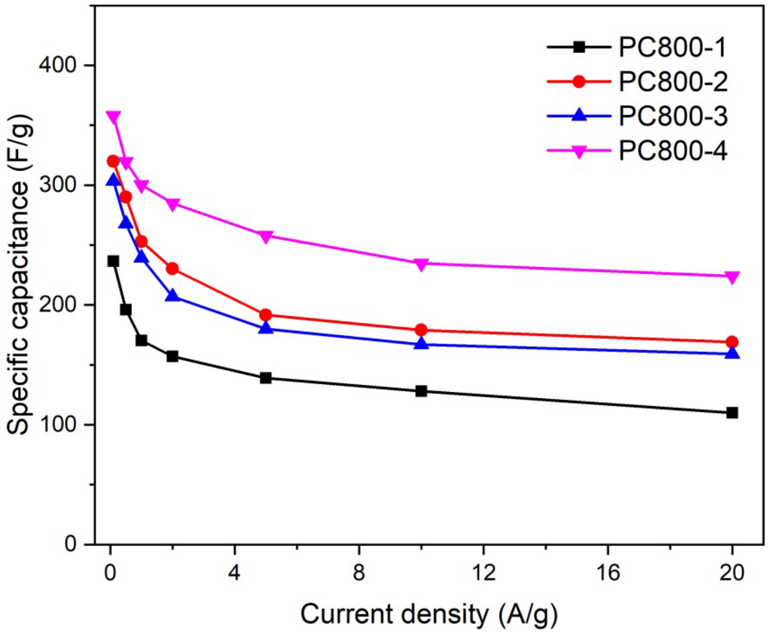


**Figure S4.** Specific capacitances of the PC800s at different current densities (0.1–20 A $g^{-1}$).
